# Endoscopic submucosal dissection using ultrathin endoscope for early gastric cancer progressing from pyloric ring to bulb

**DOI:** 10.1055/a-2264-1164

**Published:** 2024-03-01

**Authors:** Takeshi Yasuda, Osamu Dohi, Naoto Iwai, Hiromu Kutsumi, Yoshito Itoh

**Affiliations:** 1 614269 Molecular Gastroenterology and Hepatology, Graduate School of Medical Science, Kyoto Prefectural University of Medicine, Kyoto, Japan; 2 38056 Department of Gastroenterology, Akashi City Hospital, Akashi, Japan

Endoscopic submucosal dissection (ESD) for early-stage gastric cancer (GC) has been widely adopted. However, GC’s progression from the pyloric ring to the duodenal bulb remain challenging. In the antegrade approach, treating the anal side is difficult, whereas in the retroflex approach, the narrow space of the duodenal bulb poses a risk of perforation when standard-diameter scopes are used 1 2 . Recently, ESD with ultrathin endoscopes has been reported 3 ; moreover, devices suitable for procedures with ultrathin endoscopes have become commercially available 4 . We report an efficient and safe ESD for GC that extended into the duodenal bulb by combining the aforementioned devices.

A 73-year-old man was referred to our hospital to treat a depressed-type early GC ( Video 1 ). The lesion measured 15 mm and extended from the anterior wall of the pyloric ring to the duodenum bulb ( Fig. 1 **a** ). An ultrathin scope (EG-L580NW; Fujifilm Co, Tokyo, Japan) with an ultrathin tip hood (Nichendo, Fujifilm) was utilized to perform ESD for this lesion ( Fig. 1 **b** ). We utilized a thin resection device (Endosaber Fine; SB Kawasumi Co, Ltd, Tokyo, Japan.) to initiate a semi-circular incision from the anal side with a retroflex view ( Fig. 1 **c** ). Next, a partial incision was made on the oral side, and the submucosal layer was dissected with an antegrade view using the pocket creation method (PCM) 2 . A total circumferential incision was made. A clip with a thread was attached on the oral side 5 , and the lesion was resected en bloc ( Fig. 1 **d** ). Hemostatic forceps that could pass through the narrow-caliber scope (RAICHO 2; Kaneka Medix Corp., Osaka, Japan) were utilized to manage intraoperative bleeding.

For resecting early gastric cancer progressing from the pyloric ring to the bulb, using an ultrathin endoscope and tip hood allows endoscopic submucosal dissection with a stable view in the bulb.Video 1

**Fig. 1:**
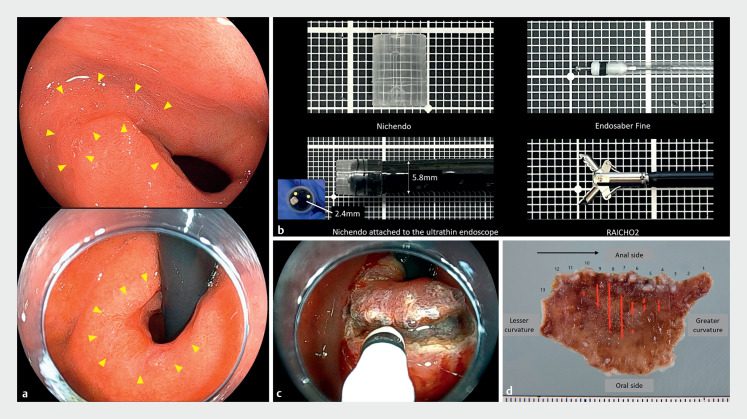
**a** Lesion seen from the antegrade and retroflex view. **b** Devices utilized. **c** Submucosal dissection of anal side with a retroflex view. **d** The resected specimen.

Utilizing an ultrathin endoscope with an ultrathin tip hood allows ESD to be performed safely and with a stable field of view, even in the narrow space of the duodenal bulb.

Endoscopy_UCTN_Code_TTT_1AQ_2AD

## Notes

Endoscopy E-Videos https://eref.thieme.de/e-videos
*E-Videos* is an open access online section of the journal *Endoscopy* , reporting on interesting cases and new techniques in gastroenterological endoscopy. All papers include a high-quality video and are published with a Creative Commons CC-BY license. Endoscopy E-Videos qualify for HINARI discounts and waivers and eligibility is automatically checked during the submission process. We grant 100% waivers to articles whose corresponding authors are based in Group A countries and 50% waivers to those who are based in Group B countries as classified by Research4Life (see: https://www.research4life.org/access/eligibility/ ). This section has its own submission website at https://mc.manuscriptcentral.com/e-videos .
